# Critical care ultrasonography in acute respiratory failure

**DOI:** 10.1186/s13054-016-1400-8

**Published:** 2016-08-15

**Authors:** Philippe Vignon, Xavier Repessé, Antoine Vieillard-Baron, Eric Maury

**Affiliations:** 1 Medical-surgical Intensive Care Unit, Limoges Teaching hospital, F-87000 Limoges, France; 2 Center of Clinical Investigation, INSERM 1435, F-87000 Limoges, France; 3 University of Limoges, Faculty of Medicine, F-87000 Limoges, France; 4 Intensive Care Unit, Section Thorax-Vascular Disease-Abdomen-Metabolism, University Hospital Ambroise Paré, Assistance Publique-Hôpitaux de Paris, 92100 Boulogne-Billancourt, France; 5 University of Versailles Saint-Quentin en Yvelines, Faculty of Medicine Paris Ile-de-France Ouest, 78280 Saint-Quentin en Yvelines, France; 6 INSERM U-1018, CESP, Team 5 (EpReC, Renal and Cardiovascular Epidemiology), UVSQ, 94807 Villejuif, France; 7 Medical Intensive Care Unit, University Hospital Saint-Antoine, Assistance Publique-Hôpitaux de Paris, Paris, France; 8 INSERM U 1136, Institut Pierre-Louis d’Epidémiologie et de Santé Publique, Paris, F-75012 France; 9 Sorbonne University, UPMC Univ Paris 06, Paris, France

**Keywords:** Echocardiography, Echocardiography Doppler, Ultrasonography, Respiratory insufficiency, Pulmonary edema, Respiratory distress syndrome

## Abstract

Acute respiratory failure (ARF) is a leading indication for performing critical care ultrasonography (CCUS) which, in these patients, combines critical care echocardiography (CCE) and chest ultrasonography. CCE is ideally suited to guide the diagnostic work-up in patients presenting with ARF since it allows the assessment of left ventricular filling pressure and pulmonary artery pressure, and the identification of a potential underlying cardiopathy. In addition, CCE precisely depicts the consequences of pulmonary vascular lesions on right ventricular function and helps in adjusting the ventilator settings in patients sustaining moderate-to-severe acute respiratory distress syndrome. Similarly, CCE helps in identifying patients at high risk of ventilator weaning failure, depicts the mechanisms of weaning pulmonary edema in those patients who fail a spontaneous breathing trial, and guides tailored therapeutic strategy. In all these clinical settings, CCE provides unparalleled information on both the efficacy and tolerance of therapeutic changes. Chest ultrasonography provides further insights into pleural and lung abnormalities associated with ARF, irrespective of its origin. It also allows the assessment of the effects of treatment on lung aeration or pleural effusions. The major limitation of lung ultrasonography is that it is currently based on a qualitative approach in the absence of standardized quantification parameters. CCE combined with chest ultrasonography rapidly provides highly relevant information in patients sustaining ARF. A pragmatic strategy based on the serial use of CCUS for the management of patients presenting with ARF of various origins is detailed in the present manuscript.

## Background

Acute respiratory failure (ARF) is a leading indication for performing critical care ultrasonography (CCUS), which combines critical care general ultrasonography (thoracic, abdominal, and vascular) and critical care echocardiography (CCE) [1]. CCUS is performed and interpreted at the patient’s bedside by the attending intensivist to establish diagnoses and guide therapeutic management [1]. In intensive care unit (ICU) patients, transthoracic echocardiography (TTE) and transesophageal echocardiography (TEE) are complementary and are routinely used according to image quality and to the clinical questions to be answered for more than two decades [2]. More recently, chest ultrasonography has provided new insights into pulmonary and pleural diseases [3]. When compared to a conventional diagnostic work-up, CCUS improves the diagnostic performance in patients sustaining ARF [4]. The present manuscript aims to review current uses of CCUS, including both CCE and chest ultrasonography, for the assessment of ICU adult patients presenting with ARF.

## Cardiogenic pulmonary edema

Cardiogenic pulmonary edema (CPE) is a frequent cause of ARF. Although congestive heart failure remains commonly encountered in this clinical setting, CPE is increasingly attributed to heart failure with a preserved ejection fraction (EF) [5]. Since CPE develops secondary to pulmonary venous congestion, its diagnosis relies on the identification of elevated left ventricular (LV) filling pressures, irrespective of systolic function. Accordingly, patients with LV diastolic dysfunction are at risk of developing CPE [6]. CCE is ideally suited to identify a causative cardiomyopathy while chest ultrasonography depicts lung and pleural consequences of fluid overload.

### Identification of elevated left ventricular filling pressures

LV filling pressures closely depend on cardiac diastolic properties and volume status. CCE assessment of LV filling pressures mainly relies on the combined use of pulse-wave Doppler interrogation of mitral inflow (early diastolic E wave and A wave during atrial contraction) and tissue Doppler measurement of LV lengthening velocity during early diastole (E’ wave) which reflects myocardial relaxation [6]. Doppler indices have been validated in ventilated ICU patients for the identification of elevated pulmonary artery occlusion pressure (PAOP) (Table 1). A restrictive mitral Doppler pattern is consistent with a PAOP >18 mmHg [7–9]. A normalized mitral Doppler profile or even an inverted E/A ratio with prolonged E wave deceleration time may reflect elevated LV filling pressures in patients with severely altered diastolic properties and significant LV hypertrophy [10]. Accordingly, normalizing E by E’ wave maximal velocity allows us to better assess LV filling pressures, especially in patients with underlying cardiomyopathy (Table 1). In the presence of regional wall motion abnormalities, the lateral and septal E’ velocities should be averaged [11]. Mitral valve disease (stenosis, regurgitation, annular calcification, valvular prosthesis) and constrictive pericarditis invalidate the use of E/E’ to assess LV filling pressure [11]. In contrast, this approach remains valid in atrial fibrillation [11] (Table 1). E/E’ accurately tracks variations in LV filling pressures in treated patients with congestive heart failure [12].

**Table 1:** Examples of Doppler indices proposed to semi-quantitatively predict left ventricular filling pressures in patients hospitalized in the intensive care unit or in the perioperative course of cardiac surgery, and in patient with atrial fibrillation (adapted from [7])

Doppler indices	Threshold values	Predicted left ventricular filling pressures	Sensitivity	Specificity	Positive predictive value
Sinus rhythm					
Mitral E/A	>2	>18 mmHg^bc^	–	–	100 %
Systolic fraction^a^	<55 %	>15 mmHg	91 %	87 %	–
	<40 %	>18 mmHg^bc^	–	–	55 %
	≤40 %	≥18 mmHg^bc^	100 %	100 %	100 %
	≤44 %	>18 mmHg^bc^	85 %	88 %	–
D wave DT	<175 ms	≥18 mmHg^b^	100 %	94 %	–
E/E’	>15	>15 mmHg^c^	86 %	88 %	–
	>7	≥13 mmHg^bc^	86 %	92 %	–
	>7.5	≥15 mmHg^bc^	86 %	81 %	–
	>9.5	>18 mmHg^bc^	100 %	86 %	–
E/Vp	>2	≥13 mmHg^bc^	–	–	–
	>2.6	>18 mmHg^bc^	100 %	86 %	–
Atrial fibrillation					
E wave DT	<150 ms	15 mmHg	71 %	100 %	–
	<120 ms	≥20 mmHg	100 %	96 %	–
D wave DT	>220 ms	≤12 mmHg	100 %	100 %	–
E/E’	>10	≥15 mmHg	75 %	93 %	–
E/Vp	≥1.4	>15 mmHg	71 %	88 %	–

^a^VTI S wave/VTI S + VTI D waves expressed as a percentage (pulmonary venous Doppler)

^b^Ventilated patients

^c^Intensive care unit patients

*DT* deceleration time of pulmonary vein D wave or of mitral E wave, *E’* maximal velocity of early diastolic tissue Doppler pulse wave recorded at the level of the mitral annulus (lateral aspect), *Vp* propagation velocity of early diastolic inflow measured in the left ventricular cavity using M-mode color Doppler, *VTI* velocity-time integral

### Diagnosis of the underlying heart disease

In patients with CPE secondary to a decompensated congestive heart failure, CCE allows a comprehensive assessment of the causative cardiomyopathy. In the absence of LV systolic dysfunction and underlying cardiomyopathy, an acute volume overload should first be ruled out (e.g., fluid overload in anuric patients, acute mitral or aortic regurgitation). Severe mitral regurgitation (MR) may be challenging to identify using TTE color Doppler mapping in the presence of eccentric jets. In this case, TEE more accurately depicts the regurgitant jet which has a clockwise or anticlockwise direction within the left atrium (LA) and may preferably enter left or right pulmonary veins, resulting in unilateral or asymmetrical pulmonary edema [13]. This frequently leads to delayed appropriate diagnosis and treatment. Unsuspected chronic MR is highly prevalent in patients presenting with CPE and associated coronary artery disease, irrespective of LV systolic function [14]. Its severity may vary over time due to the dynamic interplay between tethering and closing forces, especially in the presence of abrupt changes in LV loading conditions and pharmacological interventions. The resulting increase in PAOP may contribute to CPE [15].

Heart failure with normal LVEF—and no acute volume overload—is increasingly encountered in patients presenting with CPE [5]. Predisposing factors including ageing, female gender, obesity, hypertension, and LV hypertrophy are frequently present in ICU patients [6]. Because of the disproportionate rise of LV filling pressures for a relatively small increase in central blood volume, LV diastolic dysfunction may lead to an abrupt increase in PAOP, and hence CPE, secondary to various precipitating factors (e.g., small blood volume expansion, hypertensive crisis, atrial fibrillation) [16]. In patients who sustain a hypertensive pulmonary edema, Gandhi et al. [5] showed that LVEF and regional wall motion score assessed with TTE were similar upon admission and after the resolution of CPE and control of blood pressure. These results suggest that hypertensive pulmonary edema is primarily related to LV diastolic dysfunction exacerbated by elevated systolic blood pressure [5]. Diagnosis of heart failure with normal EF relies on the combined presence of: (i) signs or symptoms of heart failure; (ii) LVEF >50 % with an indexed LV end-diastolic volume <97 mL/m^2^ (i.e., no significant LV enlargement); and (iii) LV diastolic dysfunction or its surrogates such as LV hypertrophy, LA enlargement, atrial fibrillation, or elevated plasma natriuretic peptides levels [17]. LV diastolic dysfunction is defined by a decreased E’ wave maximal velocity <8 cm/s and <10 cm/s at the septal and lateral side of the mitral annulus, respectively, or <9 cm/s average [16]. The severity of LV diastolic dysfunction can be graded based on Doppler parameters [11].

### Associated lung and pleural changes depicted by chest ultrasonography

**Fig. 1:**
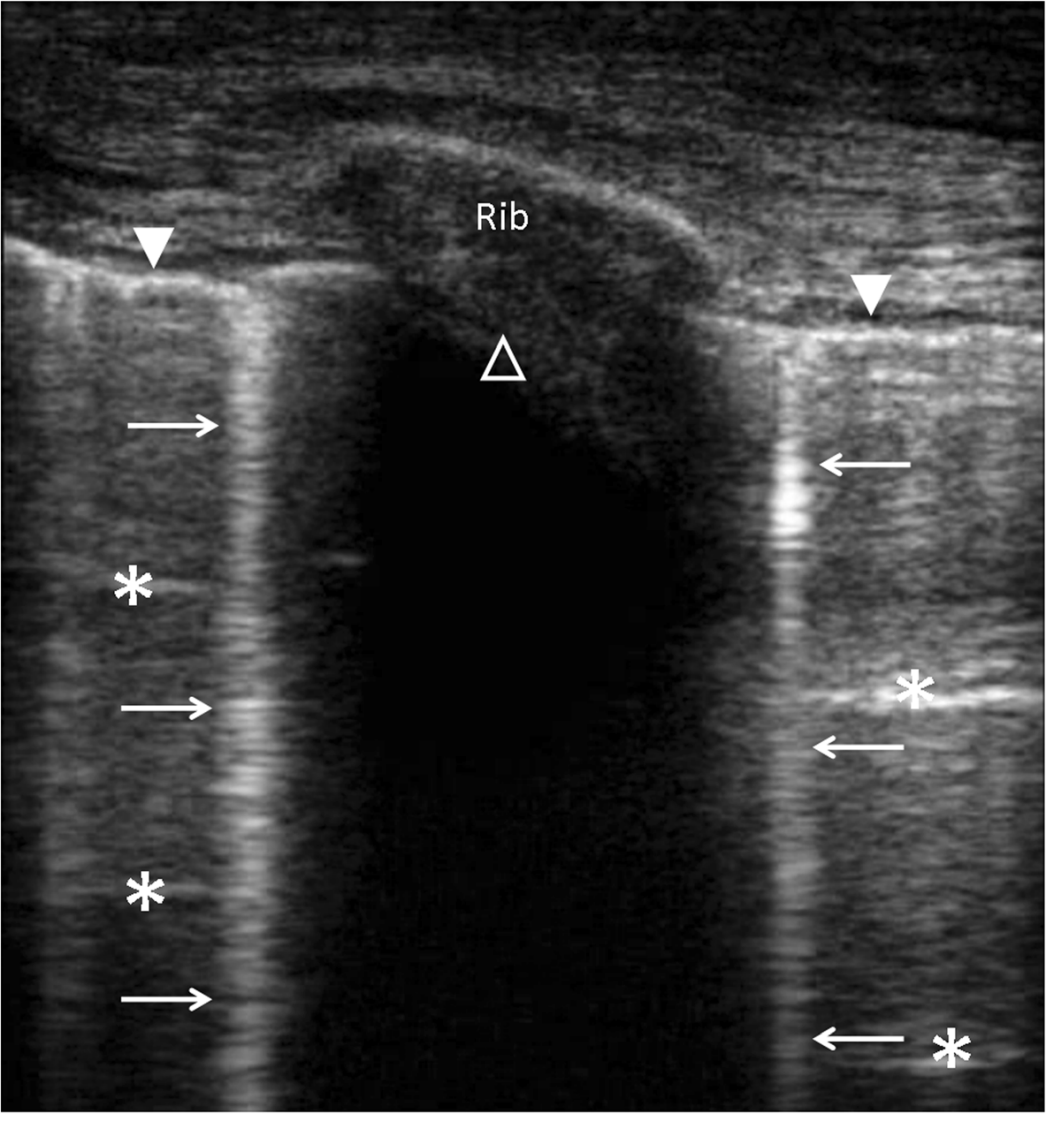
Longitudinal view of an intercostal space using chest ultrasonography disclosing the pleural line (*closed white arrow head*), A-lines (*white asterisks*), B-lines (*white arrows*), and shadowing related to the rib (*open white arrow head*)

Lung ultrasonography relies on the identification of findings (pleural effusion and parenchymal condensation) and artifacts (lung sliding, lung point, A-line, B-line) which can be combined [18, 19] (Table 2) (Fig. 1). A strict technique of examination should be used [3]. Pleural effusion is observed in more than 90 % of patients presenting with decompensated heart failure [20]. Its diagnosis relies on the presence of a free fluid collection surrounded by well-identified anatomical borders (pleural layers and diaphragm), which varies in size during the respiratory cycle [21]. The longitudinal plane allows the confirmation of the thoracic location of the effusion (i.e., above the diaphragm), whereas the transversal plane is used to quantitatively assess the volume of pleural effusion [22–25]. Transudates are typically bilateral and anechoic, whereas exudative effusions may be echoic or not, and potentially loculated. Ultrasound-guided thoracentesis decreases the risk of pneumothorax [26].

**Table 2:** Chest ultrasonographic findings associated with the main lung diseases

	Normal lung	Pneumothorax	Interstitial syndrome	Pulmonary edema^a^	Pneumonia	Pulmonary embolism	Fibrosis	Lymphangitis
A-lines	Present	Present or absent	Absent	Absent	Absent	Present when central pulmonary embolism	Absent	Absent or present
B-lines	Absent except rare B line in lower intercostal spaces	Rules out pneumothorax	Present proportional with interstitial syndrome intensity	Present +++	Present ++	Absent	Present	Present
Condensation	Absent	Absent	Absent	Present in case of massive edema	Present +++ except when pneumonia not in contact with pleura	Subpleural condensation in case of peripheral infarct	Absent	Absent
Lung sliding	Present	When present rules out pneumothorax	Present	Present	Present	Absent or present	Absent or present	Absent or present
Lung point	Absent	Pathognomic of pneumothorax	Absent	Absent	Absent	Absent	Absent	Absent
Pleural effusion	Absent	Absent	Absent	Might be present	Present+	Might be present	Absent	Might be present

^a^Irrespective of its cardiogenic origin or not

The presence of B-lines (and the loss of A-lines) is highly suggestive of interstitial pulmonary edema (Table 2). Importantly, B-lines only reflect the presence of water in the lung, although it may not help separating between cardiogenic and inflammatory causes. Separated B-lines are consistent with the presence of a moderate interstitial edema, while coalescent B-lines indicate a severe interstitial edema [27]. A consolidation aspect suggests alveolar filling [28]. Both the number and characteristics of B-lines (i.e., separated or coalescent) have been correlated with the measurement of extravascular lung water [28] and with fluid balance in anuric patients receiving ultrafiltration [29]. In mechanically ventilated patients, exclusive or predominant A-lines in anterior chest areas predict a PAOP <18 mmHg [27, 30]. Nevertheless, the diagnostic accuracy of lung ultrasonography for predicting PAOP varies among studies, presumably due to the absence of a standardized quantification of B-lines [31]. This remains a major limitation of lung ultrasonography.

### How to apply a pragmatic critical care ultrasonographic strategy in patients presenting with cardiogenic pulmonary edema

CCE should be performed as a first-line diagnostic test in patients presenting with suspected CPE. First, it allows semi-quantitative assessment of LV filling pressures to differentiate CPE from acute respiratory distress syndrome (ARDS) [32]. Second, CCE helps the front-line intensivist to identify the underlying cardiopathy or the mechanism of CPE. Interpretation of CCE examination should ascertain that observed findings fully account for the clinical presentation. Third, CCE may be used to guide and to monitor both the efficacy and tolerance of treatment in patients with CPE. For example, CCE may depict the beneficial effects of positive pressure ventilation on central hemodynamics [33], or the decrease of LV filling pressure resulting from a negative fluid balance [34]. Hypervolemia requires diuretics or ultrafiltration while titrated intravenous vasodilators may reduce LV afterload, and hence the pressure gradient between the LV and the LA, and ultimately the volume of the associated MR [35]. Finally, CCE may depict the partial or full recovery of a transiently depressed LV systolic function, as in CPE secondary to an ischemic myocardial event or induced by a stress cardiomyopathy [36].

## Ventilator weaning failure

Spontaneous breathing trial (SBT) has been compared to an exercise [37]. Interruption of positive-pressure ventilation increases venous return and LV afterload, decreases LV compliance, and may induce cardiac ischemia, all of which contribute to an abrupt increase in LV filling pressures [38]. Accordingly, patients with LV diastolic dysfunction are at high risk of developing weaning pulmonary edema because they have a reduced diastolic and chronotropic reserve, and an abnormal ventricular-arterial coupling [39].

### How to infer weaning failure from a cardiac origin

**Fig. 2:**
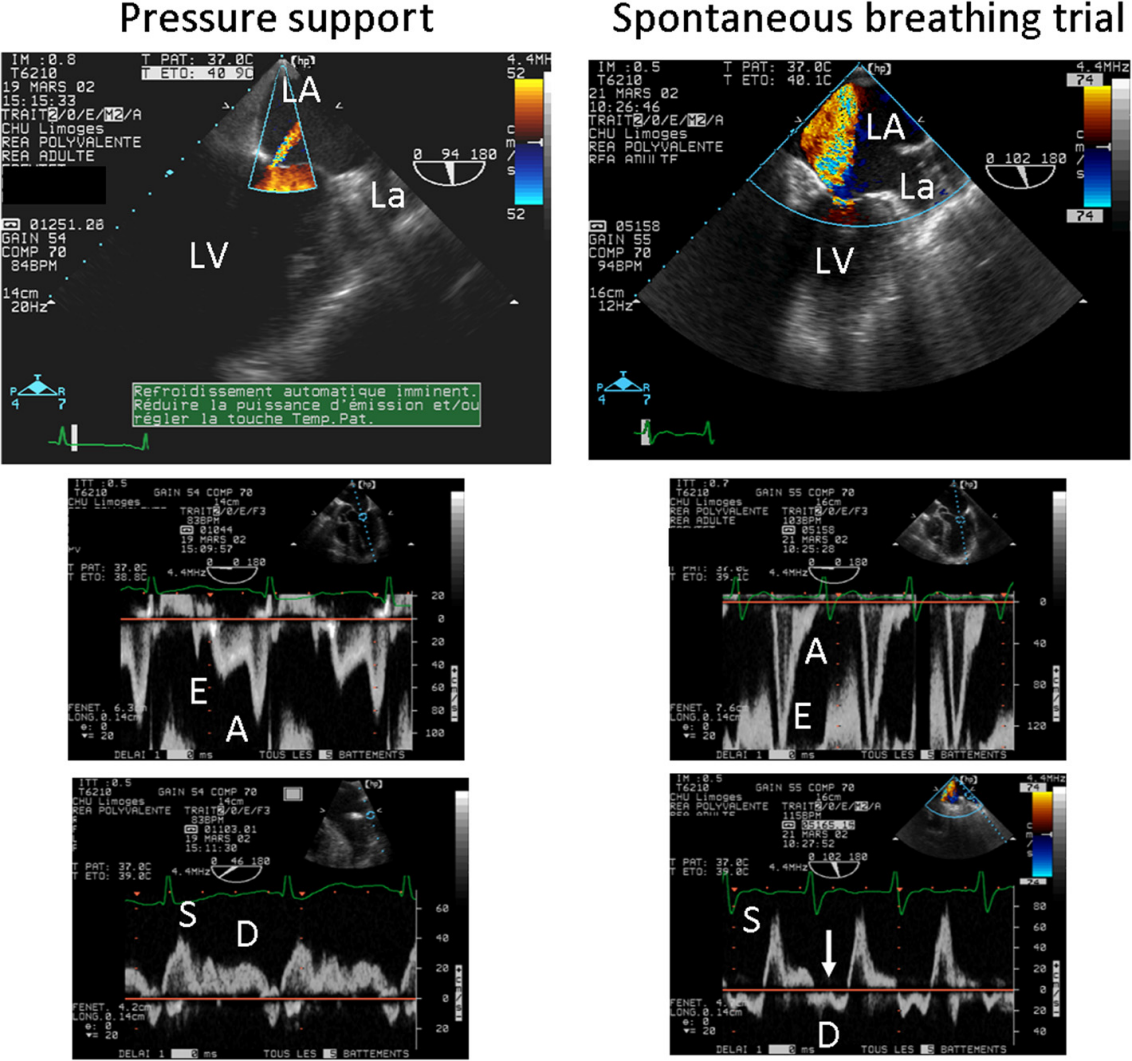
Transesophageal echocardiographic hemodynamic monitoring in a patient with a known ischemic cardiomyopathy who failed ventilator weaning. Under pressure support, a trivial mitral regurgitation was disclosed by color Doppler mapping in the two-chamber view (*upper left*), and left cardiac filling pressures were low, as reflected by an inverted mitral E/A Doppler pattern (*middle left*) and a predominant pulmonary vein S wave (*lower left*). During the spontaneous breathing trial, a severe mitral regurgitation occurred (*upper right*), mitral Doppler pattern was restrictive (*middle right*), and pulmonary vein Doppler disclosed a reversed D wave consistent with massive mitral insufficiency (*lower right*, *arrow*). The acute mitral regurgitation was attributed to a papillary muscle dysfunction secondary to a transient myocardial ischemic event. *LA* left atrium, *La* left auricle, *LV* left ventricle. Adapted from [7]

CCE accurately depicts an SBT-induced increase in LV filling pressures, irrespective of systolic function [40]. Patients who fail ventilator weaning exhibit lower LVEF and higher LV filling pressures prior to SBT [41, 42]. In addition, patients with LV diastolic dysfunction of any grade have a higher rate of SBT failure than patients with normal relaxation [43]. The site of E’ measurement (lateral, septal, or both) and values of E/E’ predictive of SBT failure differ between studies, with threshold values ranging from 7.8 to 14.5 [43–45]. Accordingly, the sensitivity and specificity of E/E’ to predict SBT failure varies from 75 % to 82 % and from 91 % to 100 %, respectively [43–45]. Such discrepancies result from the heterogeneity of study populations, including highly selected patients [44] or patients with atrial fibrillation [45]. In cardiac patients ventilated for CPE, MR is frequently depicted at baseline and its severity tends to increase during SBT [46] as a result of abrupt changes in LV loading conditions [15]. A marked increase in MR volume during SBT may result in weaning failure (Fig. 2). Adamopoulos et al. [47] reported two cases of intractable weaning pulmonary edema in patients with hypertrophic cardiomyopathy associated with a systolic anterior motion of the mitral valve which resulted in a dynamic LV outflow tract obstruction and severe MR. Noticeably, right heart catheterization erroneously related weaning failure to a congestive heart failure while CCE corrected the diagnosis. Accurate identification of such complex mechanisms of weaning failure is crucial since symptomatic treatment of congestive heart failure (i.e., diuretics, vasodilatators, inotropes) may be detrimental in this setting and beta-blockers should be considered [47]. During SBT using a T-tube [40, 41, 44], E/E’ values tend to be higher than during a 7-cmH_2_O pressure-support with zero positive end-expiratory pressure (PEEP) trial [45, 46], the difference being not significant in a small study population [48]. Whether T-tube SBT is more sensitive to trigger a weaning pulmonary edema in high-risk patients remains to be established in larger series. Taken together, these studies suggest that LV diastolic dysfunction is strongly associated with weaning failure and that CCE allows the identification of high-risk patients and helps predict weaning failure of cardiac origin.

### Use of chest ultrasonography in weaning failure

During SBT, the inability to maintain lung aeration is consistent with weaning failure. The development or increased number of B-lines reflects this loss of lung aeration. Soummer et al [49] used a standardized evaluation of lung aeration (Lung Ultrasound Score (LUS)) by assessing the aeration for each hemi-thorax as follows: upper and lower parts of anterior, lateral and posterior area of the lung (12 areas). For a given region of interest, each intercostal space was scanned and a number of points was allocated according to the most severe abnormality: normal aeration (lung sliding with A-lines or less than two isolated B-lines; 0 points), moderate loss of aeration (3 or more separated B-lines; 1 point), severe loss of aeration (coalescent B-lines/curtain sign; 2 points), and lung consolidation (3 points). The LUS was calculated as the sum of points and ranged between 0 and 36 points. Among 100 patients undergoing SBT, the increase in LUS (measured before and at the end of SBT) was greater in patients who failed the trial (*n* = 14) when compared to those who succeeded. This suggested a greater loss of lung aeration in patients who failed SBT [49]. Interestingly, in 86 patients who had been extubated, the LUS measured at the end of SBT was greater in patients who experienced post-extubation respiratory distress (*n* = 29) and remained higher 4 h after extubation. A LUS ≥17 measured at the end of the SBT was associated with a likelihood ratio of post-extubation respiratory distress of 11.8 [49]. Finally, large pleural effusions may participate in failure to be weaned from the ventilator.

### How to apply a pragmatic critical care ultrasonographic strategy in patients at high risk of weaning failure

CCE should be used as a screening tool to objectively identify patients at high risk of weaning failure [41–43]. In these patients, LV filling pressures should be precisely determined prior to and during SBT to depict any significant increase in PAOP [44]. The absence of a significant SBT-induced increase of E/E’ and of systolic pulmonary artery pressure in an asymptomatic patient allows consideration of extubation with a low risk of failure. In contrast, a clinical failure of SBT should trigger prompt CCE assessment to depict its precise mechanism since a cardiac origin is frequent, especially in early failures [47]. This allows tailoring of medical therapy to better prepare the high-risk patient for a new SBT [6].

## Acute respiratory distress syndrome

ARDS is an injury of both the alveoli and the pulmonary circulation, leading to acute pulmonary hypertension. With its ability to evaluate lung aeration, pulmonary artery pressure, and right ventricular (RV) function, CCUS is ideally suited to guide the management of ARDS patients. Specifically, it provides valuable information on the effects of PEEP on lung aeration and RV function. In patients with suspected ARDS, the first step is to rule out a cardiac failure or a fluid overload as the origin of ARF [50]. CCE is best suited for providing the hemodynamic criterion of ARDS in accurately assessing LV filling pressures [51].

### Why perform critical care echocardiography in ARDS patients?

Circulatory failure involves more than half of ARDS patients [52, 53]. Among the most severe patients, those with moderate-to-severe ARDS according to Berlin’s definition [50], two-thirds to three-fourths of them require the administration of catecholamines [54–57] over a mean period of 10 days [52, 58]. In most cases, shock is related to sepsis-induced hypovolemia and vasoplegia which are accurately depicted by echocardiography [59]. Nevertheless, ARDS induces specific circulatory changes which result from an uncoupling between the RV and the pulmonary circulation, and may lead to RV failure appearing as an acute cor pulmonale (ACP). Its prevalence reached 22 % in a recently reported series exceeding 700 patients [56]. This RV failure is due to the conjunction of acute pulmonary hypertension related to ARDS [60, 61] and the deleterious effects of mechanical ventilation [62, 63], even with the use of “protective” settings. ACP is defined by the association of RV dilatation and a paradoxical septal motion [64]. It can easily be diagnosed using TTE, but TEE should be preferred since it is less operator-dependent and more reproducible when ARDS patients are sedated and under mechanical ventilation [65], even in the prone position [66]. Patients can also be monitored using smaller, less invasive TEE probes. Vieillard-Baron et al. [67] reported that a single-use miniaturized probe (diameter 5.5 mm) could be useful in helping management decisions in ventilated patients with hemodynamic failure. Specifically, this TEE probe may allow the diagnosis and follow-up of ACP by a strict qualitative single-plane two-dimensional image monitoring. Another miniaturized TEE probe (diameter 5.5 mm) with multiplane imaging and Doppler capabilities has also been successfully used in ventilated ICU patients with cardiopulmonary compromise [68]. However, the clinical value of these new miniaturized TEE probes has to be confirmed in ARDS patients. In the most severe cases, RV failure may be suspected in the presence of significant pulse pressure variations [69], which are due to afterload-related respiratory variations of RV ejection flow. These variations can be detected using the pulsed wave Doppler into the RV outflow track or the main pulmonary artery [70]. Usually, ACP occurs within the first 3 days of mechanical ventilation [71]. Severe ACP (defined as RV and LV end-diastolic ratio ≥1 in conjunction with a paradoxical septal motion) occurs in less than 10 % of ARDS patients, but is strongly associated with in-hospital mortality [56]. Four risk factors of ACP, irrespective of the severity, have been reported: pneumonia as a cause of ARDS, PaO_2_/FiO_2_ ratio <150 mmHg, driving pressure ≥18 cmH_2_O, and PaCO_2_ ≥ 48 mmHg. When all these factors are present, the risk of ACP exceeds 60 %, whereas when no factor is present the risk is lower than 10 % [56].

### How to apply a pragmatic critical echocardiographic strategy during ARDS

CCUS is usually part of the initial assessment of most ICU patients. Specifically, the initial CCE evaluation of patients with ARDS is best performed within 24 h of ICU admission or tracheal intubation to achieve three objectives. First, it allows intensivists to rule out any cardiogenic mechanism at the origin of ARF (see above). Second, it provides a comprehensive assessment of hemodynamics and potentially a better understanding of the mechanism of a potential associated circulatory failure, especially in case of sepsis [59]. In some patients, ACP may already be diagnosed. Third, it rules out any significant right-to-left shunting across a patent foramen ovale, especially when the level of hypoxemia seems not to be completely explained by alveolar damage, or when increasing PEEP induces unexpected deterioration of the PaO_2_/FiO_2_ ratio [72]. At the end of initial TEE examination, two distinct groups of ARDS patients are identified. The first group is composed of hemodynamically stable patients with no more than one identified risk factor for ACP. Then, CCE could be only considered in case of circulatory failure, unexplained hypoxemia, or significant pulse pressure variation. The second group is represented either by hemodynamically unstable patients or by patients with at least two risk factors for ACP [56]. In these patients, CCE could be routinely performed, at least once a day until significant respiratory improvement, to potentially detect findings consistent with RV failure. This could lead to the adaptation of respiratory settings and help the decision of performing prone ventilation in certain patients.

### Critical care echocardiography-guided ventilator management

Most of the adaptation of the respiratory management has been described in the so-called “RV protective approach” [73]. Briefly, further decreasing tidal volume allows limiting the driving pressure in a safe range, whereas increasing the PEEP is still questionable, although it also limits the driving pressure. It is probably beneficial in situations where the lung is largely de-recruited [74], but it could be detrimental when inducing overdistension in patients with a low potential of recruitment [75, 76]. In all cases, PEEP titration should be adapted to RV tolerance evaluated by CCE. In the most severe patients, it is sometime impossible to limit driving pressure, PEEP, and PaCO_2_ on the one hand and to maintain a PaO_2_/FiO_2_ ratio in a “safe” range on the other hand. In such a situation, the prone position is valuable in the absence of any contraindication since it has been proven to increase oxygenation and compliance of the respiratory system, and to decrease PaCO_2_ without increasing the PEEP level [77]. Interestingly, the prognostic improvement observed in the prone ventilation group of the PROSEVA study [54] was not related to changes of blood gas analysis [78], and prone ventilation was associated with more days without cardiovascular compromise [54]. As expected, the prone position enables normalizing RV function in patients with ACP [79]. Whether a strategy of driving pressure reduction associating ultraprotective mechanical ventilation with CO_2_ extracorporeal removal could improve RV function and prognosis in ARDS patients remains to be elucidated [80].

### Additional value of chest ultrasonography in ARDS patients

Systematic ultrasonographic examination of pleural spaces allows the ruling out of the presence of large pleural effusions which may participate in chest X-ray opacities, thus contributing to establishing the diagnosis of ARDS [50]. In ARDS patients, the aim of recruitment maneuvers is to improve lung aeration of non- or poorly aerated lung areas. Using lung ultrasonography, Bouhemad et al. [81] assessed the impact of recruitment maneuvers on lung aeration. For each area, the change in aeration induced by the recruitment maneuver was quantified. The change in aeration of the 12 areas of interest was summed and this defined the ultrasound reaeration score. The authors demonstrated that lung recruitment measured by pressure-volume curves and ultrasound reaeration score were closely correlated (Rho, 0.88; *p* < 0.0001). They also observed that an ultrasound reaeration score ≥18 predicted a gain of aeration of more than 600 mL. Conversely, an ultrasound lung reaeration score <14 indicated an aeration gain between 75 and 450 mL. The reaeration score and observed increase in PaO_2_ were significantly correlated (Rho, 0.63; *p* < 0.05) [81]. As previously suggested by computed tomography (CT) scan studies [82], alveolar recruitment was predominantly observed in poorly aerated regions located in the anterior and lateral parts of the lung. In contrast, reaeration of consolidation was infrequent [81].

## Other causes of acute respiratory failure

### Acute pulmonary embolism

Patients sustaining pulmonary embolism (PE)-related RV failure commonly present with a circulatory failure, rather than with isolated ARF. In this clinical setting, CCE may expedite diagnosis at the bedside in depicting ACP in conjunction with an embolus-in-transit within right cardiac cavities or with a thrombus entrapped into the proximal pulmonary arteries [83]. Vascular ultrasonography allows the identification of clinically silent deep vein thrombosis. Fibrinolysis is recommended in patients with obstructive shock and should be discussed in hemodynamically stable patients with echocardiographically documented RV failure and troponin increase.

A-line pattern associated with lung sliding and normal lung surface, especially when associated with a non-compressible deep vein, is highly suggestive of central PE. In a population of 352 patients with suspected PE (194 patients with confirmed diagnosis), the presence in the dorsal and basal areas of at least two triangular or rounded pleural-based lesions or one typical lesion associated with pleural effusion had a 74 % sensitivity and a 95 % specificity for the diagnosis of distal PE [84].

### Pneumonia

CCE allows hemodynamic assessment of patients sustaining severe pneumonia associated with septic shock. Chest ultrasonographic findings associated with infectious pneumonia are lung consolidation and pleural effusion. The presence of lung consolidation has a 93 % sensitivity to identify community-acquired pneumonia in non-severely immunosuppressed patients [85]. An air bronchogram is observed in 70 % to 97 % of the cases, and is highly suggestive of pneumonia when it appears to be dynamic [86], since it is not associated with resorption atelectasis [87]. Thoracic ultrasonography is more sensitive than CT scan to identify the evolution of consolidation towards sequestration [88]. The accuracy of B-lines for the diagnosis of community-acquired pneumonia remains controversial. Conversely, B-lines associated with lung consolidation can be observed in ventilator-associated pneumonia. With successful antibiotic treatment, reaeration of the lung is associated with transformation of consolidation into B-lines and with the progressive regression of B-lines [89].

### Miscellaneous

Thoracic ultrasonography may provide further information in patients with ARF of other etiologies (e.g., pneumothorax, asthma, exacerbation of chronic obstructive pulmonary disease (COPD)) (Table 1). Lung sliding rules out pneumothorax. In the absence of lung sliding, a B-line pattern also excludes a pneumothorax. In the absence of B-lines, the presence of a lung point is specific of a pneumothorax but has only 60 % sensitivity [90]. When lung sliding, B-lines, and a lung point are not found, chest ultrasonography cannot confidently confirm or rule out a pneumothorax. The lung pulse has not been prospectively evaluated in this setting [31]. Patients with COPD exacerbation (not related to CPE) or asthma typically exhibit normal lung aeration reflected by a normal ultrasound pattern (A-lines with lung sliding) [91]. Overall, thoracic ultrasonography is a valuable extension of clinical examination but fails to replace it [92].

## Conclusion

CCUS combining CCE and chest ultrasonography rapidly provides valuable information in patients presenting with ARF. A pragmatic and systematic applied protocol may first evaluate how aerated is the lung and whether there is pulmonary edema. CCE helps the physician in identifying the cause of poor lung aeration or lung edema, and in assessing LV filling pressure and ventricular function. In various clinical settings, including difficult ventilator weaning, CCUS is valuable in guiding the diagnostic work-up and therapeutic management of patients with ARF, whether they are mechanically ventilated or not.

## Abbreviations

ACP, acute cor pulmonale; ARDS, acute respiratory distress syndrome; ARF, acute respiratory failure; CCE, critical care echocardiography; CCUS, critical care ultrasonography; COPD, chronic obstructive pulmonary disease; CPE, cardiogenic pulmonary edema; EF, ejection fraction; ICU, intensive care unit; LA, left atrium; LUS, lung ultrasound score; LV, left ventricular; MR, mitral regurgitation; PAOP, pulmonary artery occlusion pressure; PE, pulmonary embolism; PEEP, positive end-expiratory pressure; RV, right ventricular; SBT, spontaneous breathing trial; TEE, transesophageal echocardiography; TTE, transthoracic echocardiography
